# Sequences Flanking the Gephyrin-Binding Site of GlyRβ Tune Receptor Stabilization at Synapses

**DOI:** 10.1523/ENEURO.0042-17.2018

**Published:** 2018-02-19

**Authors:** Nora Grünewald, Audric Jan, Charlotte Salvatico, Vanessa Kress, Marianne Renner, Antoine Triller, Christian G. Specht, Guenter Schwarz

**Affiliations:** 1 Department of Chemistry, Institute of Biochemistry, University of Cologne, Cologne 50674, Germany; 2 École Normale Supérieure, Centre National de la Recherche Scientifique, Institut National de la Santé et de la Recherche Médicale, Institute of Biology (IBENS), Paris Sciences et Lettres Research University, Paris 75005, France; 3 Institut du Fer à Moulin (IFM), Université Pierre et Marie Curie, Paris 75005, France; 4 Center for Molecular Medicine Cologne (CMMC), University of Cologne, Cologne, Germany; 5 Cologne Excellence Cluster on Cellular Stress Responses in Aging-Associated Diseases (CECAD), University of Cologne, Cologne, Germany

**Keywords:** bimodal binding, binding site, gephyrin, glycine receptor, receptor clustering

## Abstract

The efficacy of synaptic transmission is determined by the number of neurotransmitter receptors at synapses. Their recruitment depends upon the availability of postsynaptic scaffolding molecules that interact with specific binding sequences of the receptor. At inhibitory synapses, gephyrin is the major scaffold protein that mediates the accumulation of heteromeric glycine receptors (GlyRs) via the cytoplasmic loop in the β-subunit (β-loop). This binding involves high- and low-affinity interactions, but the molecular mechanism of this bimodal binding and its implication in GlyR stabilization at synapses remain unknown. We have approached this question using a combination of quantitative biochemical tools and high-density single molecule tracking in cultured rat spinal cord neurons. The high-affinity binding site could be identified and was shown to rely on the formation of a 3_10_-helix C-terminal to the β-loop core gephyrin-binding motif. This site plays a structural role in shaping the core motif and represents the major contributor to the synaptic confinement of GlyRs by gephyrin. The N-terminal flanking sequence promotes lower affinity interactions by occupying newly identified binding sites on gephyrin. Despite its low affinity, this binding site plays a modulatory role in tuning the mobility of the receptor. Together, the GlyR β-loop sequences flanking the core-binding site differentially regulate the affinity of the receptor for gephyrin and its trapping at synapses. Our experimental approach thus bridges the gap between thermodynamic aspects of receptor-scaffold interactions and functional receptor stabilization at synapses in living cells.

## Significance Statement

The number of receptors at a synapse defines the strength of signal transmission and is directly dependent on the binding of the receptors to scaffold proteins beneath the synaptic membrane. In this study, we discovered the molecular basis for a dual-affinity interaction between glycine receptors (GlyRs) and gephyrin scaffolds. We identified GlyR sequences that are specifically required for high- and low-affinity binding. Using single-molecule tracking in cultured neurons both sites were shown to act as elements regulating the diffusion and trapping of GlyRs as a result of the altered receptor-scaffold binding. The novelty of our approach lies in the unique combination of biochemical data of purified proteins with single molecule diffusion analysis. It exemplifies that binding properties can be extracted by analyzing the diffusion behavior of molecules in living cells.

## Introduction

The amount of neurotransmitter receptors at synapses determines the strength of synaptic transmission. While the receptors diffuse laterally in the neuronal plasma membrane due to Brownian motion, their accumulation at synapses is a consequence of the transient binding to postsynaptic scaffold proteins (Dahan et al., 2003; Choquet and Triller, 2013). The molecular processes underlying the receptor-scaffold interaction can thus shed light on the diffusion-trapping mechanism at synapses and its implication in the regulation of synaptic transmission (Choquet and Triller, 2003; Petrini and Barberis, 2014).

Gephyrin is the major scaffolding molecule at inhibitory synapses, providing a platform for the immobilization of glycine receptors (GlyRs) and GABA type A receptors (GABA_A_Rs; Kirsch et al., 1993; Tyagarajan and Fritschy, 2014). Gephyrin is a multifunctional protein that catalyzes a metabolic reaction in all tissues (Fritschy et al., 2008) in addition to its receptor clustering function in neurons. Both GlyRs and GABA_A_Rs belong to the pentameric family of ligand-gated ion channels (pLGICs), in which each subunit consists of an enlarged N-terminal ligand binding domain followed by four transmembrane domains (TMs; Corringer et al., 2012). The third and fourth TMs are connected via large intracellular loops (ILs) that bind to the C-terminal E-domain of gephyrin with subunit-specific affinities (Sola et al., 2004; Tretter et al., 2008, 2011; Maric et al., 2011, 2014a; Specht et al., 2011; Kowalczyk et al., 2013). In the case of the GlyR and gephyrin binding is mediated by the β-subunit (Meyer et al., 1995; Patrizio et al., 2017). Consequently, gephyrin depletion completely abolishes the synaptic clustering of GlyRs (Levi et al., 2004; Zacchi et al., 2008), which in turn leads to severe encephalopathy due to the failure in inhibitory neurotransmission (Dejanovic et al., 2015).

Given the importance of GlyR-gephyrin binding for inhibitory signal transmission in the spinal cord and the brainstem, the molecular interaction between the receptor and scaffold proteins has been subject to detailed biochemical characterization. These studies disclosed a two-site or bimodal binding mechanism based on two different binding affinities (Schrader et al., 2004; Sola et al., 2004; Herweg and Schwarz, 2012). However, the molecular determinants of this bimodal binding and their specific effects on receptor trapping at synapses have not been identified. In this study, we have applied a biochemical approach to identify the molecular determinants that underlie the two-site binding mechanism between the GlyR β-loop and gephyrin. Using single-particle tracking in living rat spinal cord neurons, we could show that the high- and low-affinity interactions regulate receptor diffusion, demonstrating that bimodal binding is a central property coordinating GlyR trapping at inhibitory synapses. The combination of biochemical data and diffusion parameters revealed a close correspondence between the two approaches, highlighting the potential of diffusion measurements to access thermodynamic parameters of interacting molecules in living cells (as proposed by Masson et al., 2014).

## Materials and Methods

### *Escherichia coli* expression constructs

The β-loop (βL) sequence encompassing amino acid residues 378–426 of the GlyRβ subunit fused to intein (pTYB2) was used as the wild-type construct (Schrader et al., 2004). Truncation variants of the βL were generated via PCR to generate fragments spanning 378–413 and 394–426, corresponding to βL-LO and βL-HI, respectively. The core βL peptide (394-413) was purchased from Pepnome (USA) with a purity of 96.72%. The residues Asp407 and Phe408 of the βL were exchanged to Pro and Gly via site-directed mutagenesis of the wild-type sequence. Chemical crosslinking and ITC interaction studies were performed with recombinant full-length gephyrin rC4 or GephE, cloned into pQE80 vector (Belaidi and Schwarz, 2013). The βL variants, full-length gephyrin and GephE were expressed in the *E. coli* strain BL21 and purified according to the protocol described below (Schrader et al., 2004; Dejanovic et al., 2015).

### Purification of recombinant proteins from *E. coli*

Intein-fused βL variants were affinity purified in lysis buffer containing 300 mM NaCl, 50 mM Tris/HCl pH 8.0, 1 mM EDTA and protease inhibitors (Complete, Roche) via the IMPACT system (New England Biolabs) according to Schrader et al. (2004). Cleavage of the βL variants was induced by the addition of cleavage buffer (50 mM NaCl, 100 mM Tris/HCl pH 7.5, 1 mM EDTA) containing 50 mM DTT as thiol and incubated for 24 h at room temperature. Cleaved βLs were separated from larger proteins and enriched using semi-permeable cellulose membrane containing devices with decreasing cutoff (10, 3 kDa; Millipore). Buffer was exchanged to ITC measurement buffer (10 mM Tris/HCl pH 8.0, 250 mM NaCl, and 1 mM β-mercaptoethanol) via dialysis over night at 4°C. After expression in *E. coli*, full-length gephyrin and GephE were purified by Ni-NTA chromatography in 300 mM NaCl and 30 mM Tris/HCl pH 8.0, containing protease inhibitors and 10 or 25 mM imidazole, respectively. For gephyrin, additional washing steps were performed with an increasing imidazole gradient (45–60 mM imidazole) and a final concentration of 250 mM imidazole for elution. Gephyrin was additionally purified by size exclusion chromatography (Superdex 16/600 prep grade) in ITC measurement buffer. GephE was further enriched via anion exchange chromatography (SourceQ15, buffer A: 50 mM Tris/HCl pH 8.0; buffer B: 50 mM Tris/HCl pH 8.0, and 1 M NaCl), followed by buffer exchange (ITC measurement buffer). All buffers for gephyrin and GephE purification were additionally supplied with 5 mM β-mercaptoethanol.

### Lentivirus expression constructs and virus production

Single molecule imaging was conducted in cultured spinal cord neurons using membrane constructs in which the β-loop of GlyRβ (N334-A455) was fused at its C terminus to a single TM of syntaxin and an extracellular Dendra2 fluorophore (derived from βL^wt^-TMD-pHluorin; Specht et al., 2011). Deletions and point mutations were introduced into the β-loop by site-directed mutagenesis (variants TMD-βL-HI: ΔV378-L393; TMD-βL-ΔCore: ΔD397-L410; TMD-βL-D407P/F408G; gephyrin binding-deficient TMD-βL-geph^-^: F398A/I400A). The coding sequences of the βL-TMD-Dendra2 variants were transferred into the pFUGW replicon (Lois et al., 2002) for lentivirus production (FU-βL-TMD-Dendra2 constructs).

To study the behavior of full-length GlyR complexes, we generated a lentivirus construct driving the expression of mEos4b-tagged GlyRβ (construct FU-mEos4b-hGlyRβ-bis, derived from FU-SP-myc-Dendra2-GlyRβ; Patrizio et al., 2017). The mEos4b sequence (Addgene) with a C-terminal SGGTGKEKS spacer was inserted after the signal peptide (SP) sequence (between residues S26 and S27 taking into account the SP) of full-length human GlyRβ (UniProt ID P48167-1) and transferred into the pFUGW vector. The following deletions and point mutations were introduced into the wild-type β-loop: variants GlyRβ-HI (ΔV378-L393); GlyRβ-LO (ΔD414-L426); GlyRβ-D407P/F408G; GlyRβ-geph^-^ (F398A/I400A).

Lentivirions were produced in HEK293 cells that were co-transfected with the replicon, pMD2.G and pCMV-dR8.74 plasmids (Addgene) using Lipofectamine 2000 (Invitrogen) and cultured at 32°C and 5% CO_2_ in neurobasal medium containing glutamax and 2% B-27. The medium was changed after 24 h and harvested at ∼50 h after transfection, passed through a filter with a pore size of 0.45 µm and stored at –80°C.

### Chemical crosslinking

The βL and gephyrin interacting sites were analyzed by chemical crosslinking using 1-(3-dimethylaminopropyl)-3-ethylcarboiimide HCl (EDC; ThermoScientific) as described previously with minor modifications (Havarushka et al., 2014). Experiments were performed as suggested in the data sheet (ThermoScientific) and the reaction stopped with 50 mM Tris/HCl, pH 8.0. Samples of crosslinked proteins were separated via 6% SDS-PAGE, followed by Coomassie staining. The bands at corresponding sizes were extracted for tryptic digestion and subsequent peptide mass fingerprinting (LC-MS/MS; Tobias Lamkemeyer) in the Proteomics Facility of the Cologne Cluster of Excellence in Cellular Stress Response in Aging-associated Diseases (CECAD) and performed as described in Havarushka et al. (2014).

### Isothermal titration calorimetry (ITC)

ITC measurements were performed using a VP-ITC system. Interaction analysis took place at 25°C in ITC buffer (10 mM Tris/HCl pH 8.0 or 7.4, 250 mM NaCl, and 1 mM β-mercaptoethanol) with cell concentrations of 20–28.6 µM gephyrin and 187–335 µM purified βL as ligand. Each experiment was repeated using proteins from independent preparations. Reference power was set to 5 µcal/s. The ligand was injected after an initial delay of 120 s with a stirring speed of 310 rpm using volumes of 3–5 µl/3–5 s for each of 50 injections with 240-s spacing. Raw data were analyzed using Origin 7 software.

### Circular dichroism (CD) spectroscopy

The secondary structure of βL-wt and βL-D407P/F408G peptides was analyzed and compared by CD spectroscopy in the far-UV spectrum (190–260 nm) according to the description in Havarushka et al., (2014). The buffer was exchanged to 100 mM NaCl, 50 mM sodium-phosphate buffer pH 7.0. CD spectra were recorded from 190–260 nm in a 0.1 cm light path quartz cuvette at 20°C with a scanning speed of 10 nm/min using a J-715 CD spectropolarimeter (Jasco). Each spectrum was recorded five times and averaged before each measurement. For background subtraction, a buffer baseline was additionally recorded and subtracted from the sample spectrum. In respect to the molecular weight [M_r_ (Da)], the number of amino acids (n), the protein concentration [mg/ml] and the path length of the cuvette [l (cm)], the measured ellipticity θ in millidegrees was converted to mean residue ellipticity [θ] in deg * cm^2^ * dmol^−1^ using the following formula: [θ] = θ * M_r_/10 * (n−1) * c * l. The mean residue ellipticity [θ] was plotted against the respective wavelength.

### Cell culture

Spinal cord dissociated neuron cultures were prepared from Sprague Dawley rats of either sex at embryonic day 14 (Specht et al., 2013), in accordance with the guidelines of the French Ministry of Agriculture and the Direction Départementale des services vétérinaires de Paris (École Normale Supérieure, animalerie des rongeurs, license B 75-05-20). Neurons were plated on glass coverslips at a density of 60,000/cm^2^ in neurobasal medium containing glutamax, 2% B-27, 5 U/ml penicillin and 5 mg/ml streptomycin (Invitrogen) and grown at 36°C and 5% CO_2_. Neurons were generally infected with lentivirus on the first day *in vitro* (DIV) and used for experiments on DIV 10–14.

### Live imaging

Spinal cord neurons expressing βL-TMD-Dendra2 variants were imaged in MEM medium without phenol red (Invitrogen), supplemented with 33 mM glucose, 20 mM HEPES, 2 mM glutamine, 1 mM sodium pyruvate, and 2% B-27. Neurons expressing full-length mEos4-GlyRβ variants were imaged in Tyrode’s solution (120 mM NaCl, 2.5 mM KCl, 2 mM CaCl_2_, 2 mM MgCl_2_, 30 mM glucose, and 25 mM HEPES; pH 7.4). Before imaging, active synapses were loaded with FM 4-64 dye (Invitrogen). Coverslips were incubated for 45 s with imaging medium containing 1 µM FM 4-64 and 40 mM KCl and rinsed. Neurons were then imaged at 35°C for up to 40 min. FM 4-64 was visualized with a mercury lamp (560-nm excitation and 684-nm emission filters) and bleached with a 561-nm laser before PALM imaging of Dendra2 or mEos4b fluorophores.

### Single particle tracking photo-activated localization microscopy (sptPALM)

SPT using PALM relies on the reconstruction of molecule trajectories by connecting the positions of moving fluorophores in consecutive image frames. Live PALM imaging was performed on an inverted Nikon Ti Eclipse microscope. The βL-TMD-Dendra2 and mEos4b-GlyRβ variants were photo-converted from the green to the red state with a 405 nm laser (100 mW) and excited using 561-nm illumination (laser output set to 200 mW). The combined laser beams were led through an optical fiber into the TIRF arm of the microscope and focused in the rear plane of a 100× immersion objective (numerical aperture 1.49). Laser intensities were adjusted with an AOTF to detect a sparse number of fluorophores in each image and to record relatively long trajectories of at least five points. This was done using pulsed 405-nm laser illumination (0.45-pms pulses during the off-time of the camera) and moderate 561-nm excitation intensity (50% of laser output). Image stacks of 10,000 frames were acquired at a frame rate of 15 ms with an Andor iXon Ultra EMCCD camera (160-nm image pixel size). The *z* position was maintained by a Nikon perfect focus system.

### Diffusion analysis

Detection and tracking of βL-TMD-Dendra2 variants were based on the MTT algorithm (Sergé et al., 2008), using a lab-made software (SPTrack v.4; Renner et al., 2017) running in Matlab (Mathworks). The center of each fluorophore signal was determined using a Gaussian fit, achieving a localization precision of ∼20 nm. Only trajectories of at least five points without blinking were retained. Trajectories were classified as synaptic when they overlapped or were within two pixels (320 nm) of the FM 4-64 positive presynaptic terminals (image binarization using multidimensional wavelet decomposition (MIA; Racine et al., 2007). Values of mean squared displacement (MSD) were calculated for each trajectory as described (Ehrensperger et al., 2007). Effective diffusion coefficients (D_eff_) were calculated by fitting the second to the fourth time point of the MSD curves against time (τ) according to the equation MSD = 4Dτ. For each trajectory of *N* ≥ 5 detections, the explored area (normalized by the number of detections) was calculated as a measure of the molecule mobility. The area was defined as the smallest convex envelope containing all the coordinates x_i_ and y_i_ of the trajectory, where 1 ≤ i ≤ N, calculated using the convex hull function in Matlab (Renner et al., 2017). The sptPALM recordings of full-length mEos4b-GlyRβ variants were done at a later stage of the project, using a similar experimental procedure, with several modifications. Recordings were background-corrected by subtracting a minimal t-projection of the raw movie from each image frame. Detection and tracking were done with a newer MTT-based analysis software (SuperRes v.1; M. Renner, unpublished). Trajectories of a minimum of six detections (five steps) were considered for analysis, allowing for blinking of one frame. D_eff_ was calculated by fitting the second to fifth time point of the MSD curve. Trajectories within a three-pixel distance (480 nm) of the FM 4-64 mask (binarized image using wavelet point detection, Icy analysis platform, Institut Pasteur; de Chaumont et al., 2012), were considered as synaptic.

### Statistical analysis

Determination of binding parameters between βL variants and full-length gephyrin or GephE via ITC was repeated using independently expressed and purified proteins. Mean binding parameters and SEM values from a minimum of three individual measurements are given in the results. An unpaired two-tailed *t* test was applied for statistical comparison via GraphPad Prism 5 and superscript letters within the results section are listed with *p* values in Table 1. The effective diffusion of TMD-βL and GlyRβ variants in spinal cord neurons was compared via one-way ANOVA (Kruskal–Wallis test). A *post hoc* Dunn’s multiple comparison test was applied due to differences in the number of detected trajectories. The comparison of membrane diffusion of TMD-βL and GlyRβ variants was based on *n* > 700 trajectories per condition from more than nine cells and three to five independent experiments (superscript letters in the results section are listed with *p* values in Table 1).

**Table 1.** Statistical analysis of ITC binding parameters between gephyrin and GlyR β-loop variants and of sptPALM data of TMD-βL and GlyRβ variants

Line	Data structure	Type of test	Power
a	Normal distribution	Unpaired two-tailed *t* test	0.0005
b	Normal distribution	Unpaired two-tailed *t* test	0.0001
c	Normal distribution	Unpaired two-tailed *t* test	0.0008
d	Normal distribution	Unpaired two-tailed *t* test	For *K*_D_ 0.3234/for ΔH 0.6921
e	Normal distribution	Unpaired two-tailed *t* test	<0.0001
f	Normal distribution	Unpaired two-tailed *t* test	<0.0001
g	Normal distribution	Unpaired two-tailed *t* test	0.0003
h	Normal distribution	Unpaired two-tailed *t* test	0.001
i	Normal distribution	Unpaired two-tailed *t* test	0.0267
j	Not normal distribution	One-way ANOVA/*post hoc* Bonferroni test	>0.05
k	Not normal distribution	One-way ANOVA (Kruskal–Wallis test)/*post hoc* Dunn’s multiple comparison test	For areas <0.001/for D_eff_ <0.001
l	Not normal distribution	One-way ANOVA (Kruskal–Wallis test)/*post hoc* Dunn’s multiple comparison test	For areas <0.001/for D_eff_ <0.001
m	Not normal distribution	One-way ANOVA (Kruskal–Wallis test)/*post hoc* Dunn’s multiple comparison test	For areas <0.001/for D_eff_ <0.001
n	Not normal distribution	One-way ANOVA (Kruskal–Wallis test)/*post hoc* Dunn’s multiple comparison test	<0.001
o	Not normal distribution	One-way ANOVA (Kruskal–Wallis test)/*post hoc* Dunn’s multiple comparison test	For areas **p**< 0.001/for D_eff_ **p**< 0.001
p	Not normal distribution	One-way ANOVA (Kruskal–Wallis test)/*post hoc* Dunn’s multiple comparison test	For areas <0.0001/for D_eff_ <0.0001

ITC-derived binding parameters from a minimum of three independent measurements were compared using an unpaired two-tailed *t* test. Diffusion values were compared via one-way ANOVA (Kruskal–Wallis test) followed by a *post hoc* Dunn’s multiple comparison test. The comparison test was applied due to differences in the number of detected trajectories (*n* = 700–10,000 synaptic trajectories for each construct from three to five independent experiments).

## Results

### Bimodal GlyR β-loop binding requires trimeric full-length gephyrin

GlyR immobilization at the postsynaptic membrane crucially depends on gephyrin. A two-site binding mechanism of the GlyR β-loop (49 residues, 378–426) has been repeatedly identified using recombinant gephyrin expressed and purified from prokaryotic or eukaryotic organisms, suggesting an intrinsic feature of the interaction (Schrader et al., 2004; Sola et al., 2004; Herweg and Schwarz, 2012; Sander et al., 2013). Full-length gephyrin expressed in *E. coli* forms stable trimers (Schrader et al., 2004; Herweg and Schwarz, 2012; Sander et al., 2013), representing its basic oligomeric form. Within a trimer, the C-terminal E-domains are in a monomeric state (Sander et al., 2013). In contrast, isolated GephE domains expressed in *E. coli* form dimers at physiologic salt concentrations and pH 7.4–8.0 (data not shown). The binding pocket for the GlyR β-loop, formed at the interface of the two monomers, has been identified in the crystal structure of the GephE dimer (Sola et al., 2004; Kim et al., 2006).

**Figure 1.**
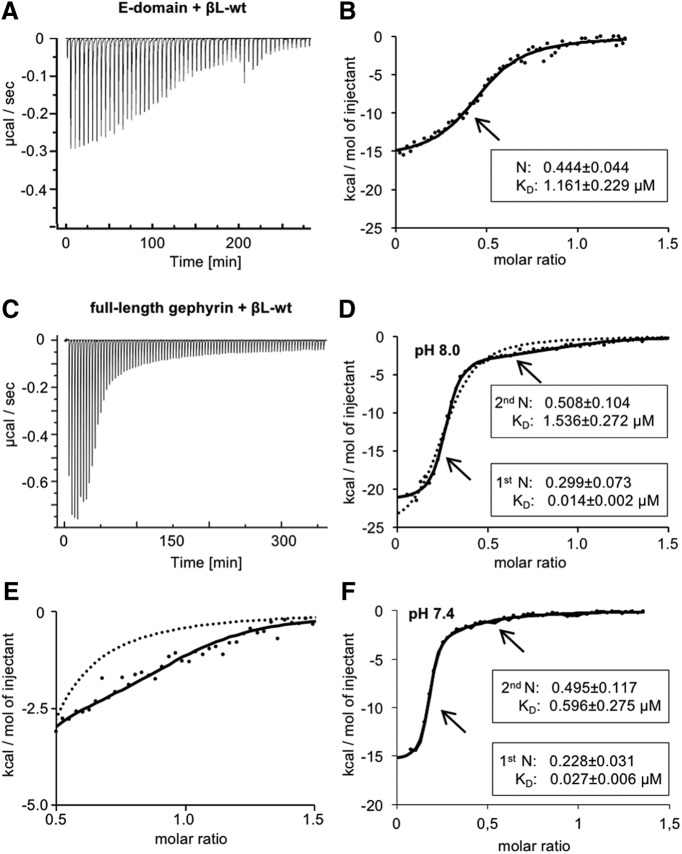
Binding properties of GlyR β-loop to full-length gephyrin or the isolated E-domain. ***A***, Representative ITC titration profile of βL-wt (378–426; 281 µM) into GephE (31 µM) at pH 8.0. The recorded peaks were corrected by baseline-corrected injection heats. ***B***, Binding isotherms (dots) of integrated binding heats were fitted to a one-site model (black line). The average dissociation constant (*K*_D_) and binding stoichiometry with GephE (N) of five independent experiments are given. ***C***, Representative ITC titration profile of βL-wt (327 µM) into gephyrin (29 µM). ***D***, Binding isotherm (dots) of integrated binding heats were fitted to a two-site model (black line) or a one-site model (dotted line). An individual measurement of βL-wt binding to gephyrin is shown alongside with averaged thermodynamic parameters of both sites (binding stoichiometry N and dissociation constant *K*_D_). Binding enthalpies (ΔH in kcal/mol) for βL-wt high and low affinity were compared using an unpaired two-tailed *t* test: *p* = 0.0005 βL-wt high-affinity site *n* = 3 versus βL-wt low-affinity site *n* = 3. ***E***, Magnification of the graph represented in ***D***, showing the fitted curves of the binding isotherm of βL-wt and gephyrin (dots) derived from the two-site (black line) or the one-site (dotted line) binding model. ***F***, ITC data showing the bimodal binding between βL-wt and gephyrin at pH 7.4. Binding isotherms of βL-wt (378–426; 248 µM) into gephyrin (28.6 µM) at pH 7.4. Binding isotherms (dots) of integrated binding heats were fitted to a two-site model (black line). An individual measurement of βL-wt binding to gephyrin is shown alongside with averaged thermodynamic parameters of both sites (binding stoichiometry N and dissociation constant *K*_D_). Binding affinities and enthalpies for βL-wt high- and low-affinity binding sites at pH 8.0 and 7.4 were compared using an unpaired two-tailed *t* test: *p* = 0.2143 *K*_D_ βL-wt high-affinity sites *n* = 3; *p* = 0.0958 *K*_D_ βL-wt low-affinity sites *n* = 3; *p* = 0.1889 ΔH βL-wt high-affinity sites *n* = 3; *p* = 0.0849 ΔH βL-wt low-affinity sites *n* = 3.

To uncover the role of dimer formation for GlyR β-loop binding, we compared the behavior of dimeric and monomeric E-domain using GephE and full-length gephyrin, respectively. The biophysical properties of the interaction with the β-loop (βL-wt, residues 378–426) were determined by ITC. GephE or full-length gephyrin were applied in the sample cell and sequentially titrated with βL-wt peptide (Fig. 1). We identified an exothermic interaction between GephE and βL-wt (Fig. 1*A*; Table 2). Unlike previous studies (Schrader et al., 2004; Sola et al., 2004), we could only fit the raw data to a one-site binding model (Fig. 1*B*). The averaged binding stoichiometry indicated a 50% occupancy of binding sites in GephE. In contrast to the interaction with dimeric E-domain, the binding isotherms of full-length gephyrin (Fig. 1*C*) were best fitted using a two-site model displaying two distinguishable binding events with βL-wt (Fig. 1*D*,*E*). Lowering the pH has been found to induce gephyrin oligomerization (Sola et al., 2004). To exclude a possible effect on βL-wt binding, we performed control experiments in which the pH was reduced from 8.0 to 7.4 (Fig. 1*F*). No significant changes in the ITC parameters were observed, confirming that high- and low-affinity binding occurs under physiologic conditions. Although both sites exhibited exothermic binding, the enthalpy reflecting the heat release differed significantly between the two sites (Fig. 1*C*; Table 3; *p* = 0.0005)^a^. This is in line with earlier data (Schrader et al., 2004;Specht et al., 2011; Herweg and Schwarz, 2012) that showed that the *K*_D_ values of high- and low-affinity sites differed by two orders of magnitude (Fig. 1*D*). The molar ratios confirmed the presence of one high- and two low-affinity sites per gephyrin trimer (Specht et al., 2011; Herweg and Schwarz, 2012) and suggested that monomeric and dimeric E-domains undergo diverging binding events with the GlyR β-loop. Bimodal binding thus appears to be a unique property of full-length gephyrin.

**Table 2.** Gephyrin E-domain binding enthalpy and binding entropy of GlyR β-loop wild-type determined by ITC

Parameter	β-Loop variant	One-site model^4^
ΔH [kcal/mol]^1^	βL-wt^3^	–16.1 ± 0.5
ΔS [cal/mol * K]^2^	βL-wt^3^	–26.8 ± 2.1

Mean values and SEM from five independent measurements.

^1^Binding enthalpy (ΔH in kcal/mol).

^2^Binding entropy (ΔS in cal/mol * K).

^3^GlyR β-loop residues 378–426.

^4^Binding isotherm fitted to a one-site interaction with gephyrin E-domain.

**Table 3.** Gephyrin binding enthalpies and binding entropies of GlyR β-loop variants determined by ITC

Parameter	β-Loop variant	Two-site model^9^		One-site model^10^
		High-affinity site	Low-affinity site	
ΔH [kcal/mol]^1^	βL-wt (pH 8.0)^3^	–19.2 ± 1.6	–2.1 ± 0.3***	–
	βL-LO^4^	–	–	–11.8 ± 2.5
	βL-Core^5^	–	–	–11.0 ± 0.6***
	βL-HI^6^	–	–	–21.5 ± 0.3***
	βL-D407P/F408G^7^	–	–	–13.8 ± 3.2
	βL-wt (pH 7.4)^8^	–14.2 ± 1.9	–4.9 ± 0.9	–
ΔS [cal/mol * K]^2^	βL-wt (pH 8.0)^3^	–28.4 ± 5.3	19.5 ± 1.4	–
	βL-LO^4^	–	–	–6.7 ± 3.5
	βL-Core^5^	–	–	–11.2 ± 2.0
	βL-HI^6^	–	–	–40.3 ± 1.1
	βL-D407P/F408G^7^	–	–	–21.7 ± 11
	βL-wt (pH 7.4)^8^	–14.2 ± 6.3	8.3 ± 4.2	–

Mean values and SEM from three or more independent measurements.

^1^Binding enthalpy (ΔH in kcal/mol).

^2^Binding entropy (ΔS in cal/mol * K).

^3^GlyR β-loop residues 378–426, pH 8.0.

^4^GlyR β-loop residues 378–413.

^5^GlyR β-loop residues 394–413.

^6^GlyR β-loop residues 394–426.

^7^GlyR β-loop residues 378–426 with substitution D407P and F408G.

^8^GlyR β-loop residues 378–426, pH 7.4.

^9^Binding isotherm fitted to a two-site interaction with gephyrin.

^10^Binding isotherm fitted to a one-site model; Data were compared using an unpaired two-tailed *t*-test: *** *p* = 0.0005 ΔH of βL-wt high-affinity site *n* = 3 vs. low-affinity site *n* = 3; *** *p* = 0.0001 ΔH of βL-wt high-affinity site *n* = 3 vs. βL-Core *n* = 9; *** *p* < 0.0001 ΔH of βL-Core *n* = 9 vs. βL-HI *n* = 4; *p* = 0.6921 ΔH of βL-Core *n* = 9 vs. βL-LO *n* = 5.

### N- and C-terminal sequences flanking the GlyR β-loop core domain tune gephyrin binding

The identification of two distinguishable binding modes of gephyrin raises the question about the nature of the bimodal interaction on the GlyR side. We therefore studied the molecular determinants within the GlyR β-loop peptide (residues 378–426) that was shown to interact with full-length gephyrin with high and low affinity.

**Figure 2.**
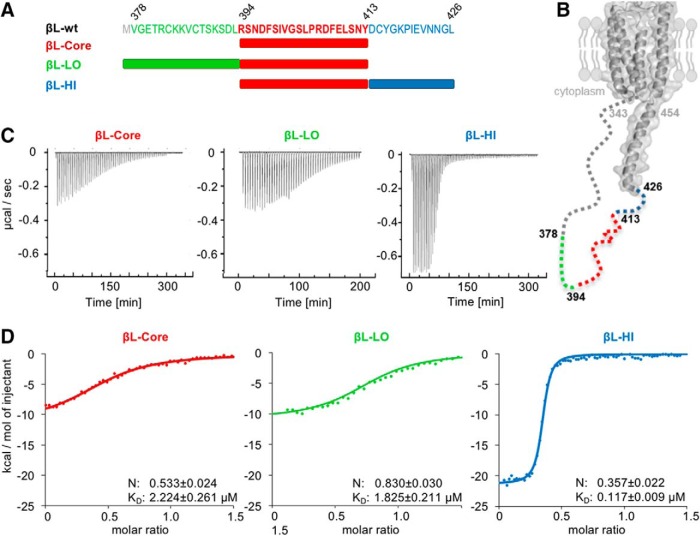
Dissection of the bimodal binding between GlyRβ and gephyrin. ***A***, GlyR β-loop peptides include full-length βL^378-426^ (βL-wt), C-terminal (βL-LO, green), and N-terminal (βL-HI, blue) truncations as well as a core region (βL-Core, red). ***B***, Structural model of the GlyR β-subunit based on the crystal structure of the nicotinic acetylcholine receptor (Unwin, 2005). Structural information corresponding to GlyRβ residues 343–426 of the ICD is lacking and therefore depicted with a dashed line. The position of the analyzed GlyR β-loop peptides is depicted. ***C***, Representative ITC titration profiles of βL-Core, βL-LO, and βL-HI (250–300 µM each) to 20–30 µM gephyrin under similar conditions. ***D***, Fitting of the ITC binding isotherms (dots) of βL-Core, βL-LO, and βL-HI to a one-site binding model (colored traces). Representative recordings are shown together with averaged *K*_D_ values and binding stoichiometry with gephyrin (N). Data were compared using an unpaired two-tailed *t* test: *p* = 0.0008 *K*_D_ of βL-wt high-affinity site *n* = 3 versus βL-Core *n* = 9; *p* = 0.3234 *K*_D_ of βL-Core *n* = 9 versus βL-LO *n* = 5; *p* < 0.0001 N of βL-Core *n* = 9 versus βL-LO *n* = 5; *p* = 0.0003 *K*_D_ of βL-Core *n* = 9 versus βL-HI *n* = 4; *p* = 0.001 N of βL-Core *n* = 9 versus βL-HI *n* = 4.

The GlyR β-loop-GephE crystal structure identified residues 398-410 as core binding sequence of the GlyR β-loop (Kim et al., 2006). We first used a 20-residue peptide (βL-Core: residues 394–413) to narrow down the residues involved in bimodal binding (Fig. 2*A*,*B*). Surprisingly, the ITC traces of βL-Core only displayed partial binding to full-length gephyrin with significantly less heat release (ΔH) as compared to the high-affinity site of the longer 49 residue loop (Figs. 1*C*,*D*, 2*C*,*D*; Table 3; *p* = 0.0001)^b^, reflecting a reduction in binding affinity (*p* = 0.0008)^c^ and the number of interacting residues. To restore high- and low-affinity interactions, either the N-terminal (βL-LO: 378-413) or C-terminal residues (βL-HI: 394–426) of the 49-residue βL-wt peptide were attached separately to βL-Core (Fig. 2*A*). Although neither the N- nor the C-terminal extensions were able to induce two-site binding with gephyrin on their own, they had pronounced effects on the binding properties of the core sequence (Fig. 2*C*,*D*). N-terminal extension of βL-Core led to a similar binding behavior to gephyrin as βL-Core alone (Table 3; Fig. 2D; *K*_D_ *p* = 0.3234, ΔH *p* = 0.6921)^d^. The binding stoichiometry, however, was significantly increased, indicating a higher occupancy of gephyrin in the presence of the N-terminal residues (Fig. 2*D*; *p* < 0.0001)^e^. The C-terminal extension of the βL-Core sequence resulted in a strong increase of heat release when titrated to gephyrin (Fig. 2*C*,*D*; Table 3; *p* < 0.0001)^f^. This increase in binding enthalpy was reflected in a significantly higher affinity (*p* = 0.0003)^g^ and a lower stoichiometry (*p* = 0.001; Fig. 2*D*)^h^, resembling the high-affinity interaction of βL-wt with gephyrin at one of the three available binding sites in trimeric gephyrin (Fig. 1*D*). These results highlight that high-affinity binding to gephyrin was significantly restored by the presence of C-terminal extension in βL-HI. A full rescue of βL-wt high-affinity binding to gephyrin requires the presence of both low- and high-affinity sites (Figs. 1*D*, 2*D* ).

Taken together, our ITC studies identified two different sequences within the GlyR β-loop that contribute to high- and low-affinity interactions with gephyrin, respectively (Fig. 2*A*). Within trimeric full-length gephyrin, each E-domain offers one binding site, of which one has a high and two have a low affinity (Fig. 1*D*; Specht et al., 2011; Herweg and Schwarz, 2012). These sequences were found to be located outside of the βL-Core region (Sola et al., 2004; Kim et al., 2006), demonstrating the impact of the flanking sequences of the GlyR β-loop for gephyrin association.

### The GlyR β-loop N terminus extends the gephyrin binding site

**Figure 3.**
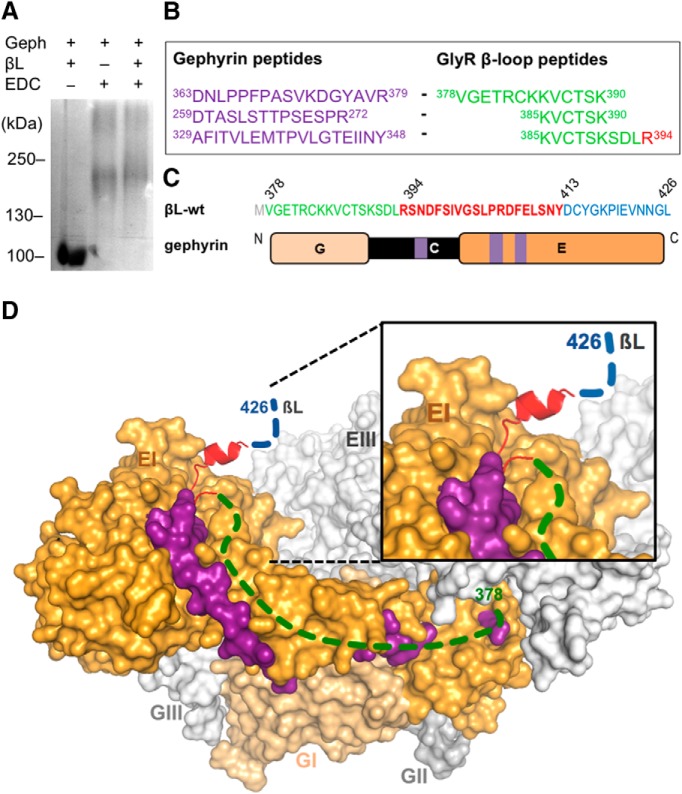
Extension of the GlyRβ binding site on gephyrin. ***A***, EDC-based crosslinking of βL-wt (378–426) and gephyrin. Gephyrin (Geph) and GlyR β-loop (βL) were treated (lanes 2 and 3) or not treated (lane 1) with EDC and separated by 6% SDS-PAGE (Coomassie staining). ***B***, Identification of crosslinked peptides. Bands corresponding to the protein complexes shown in ***A*** were extracted and analyzed by peptide mass fingerprinting. The crosslinked peptides of gephyrin (purple) and the β-loop (green, red) were identified several times. ***C***, Amino acid sequence of βL-wt with the localization of N- (green, 378–393) and C-terminal (blue, 414–426) flanking sequences of the core gephyrin-binding site (red, 394–426). Schematic representation of gephyrin domains with highlighted positions of identified peptides in the C- and E-domain (purple). ***D***, Surface representation of a modeled trimeric full-length gephyrin (modified from (Belaidi and Schwarz, 2013) with highlighted peptides identified in the crosslinked gephyrin-GlyR β-loop complex. Dashed lines indicate regions in the βL for which structural information is lacking. Gephyrin protomer II and III: light gray; protomer I: E-domain, orange and G-domain, light orange; βL core sequence: red; βL N-terminal flanking sequence: green; βL C-terminal flanking sequence: blue; and crosslinked peptides of the gephyrin E-domain: purple.

The 49-residue βL-wt comprises high- and low-affinity sites for gephyrin interaction. However, the crystal structure of the GephE-βL complex only revealed the interface of βL-Core (Kim et al., 2006). Based on our previous results, we now aimed at identifying the additional gephyrin regions that participate in the bimodal binding. To this aim, we applied a chemical crosslinking approach using EDC. Using EDC, two proteins may be fixed without a spacer, indicating a close proximity of the crosslinked residues. The purified full-length gephyrin and βL-wt peptide were incubated with EDC and subsequently subjected to SDS-PAGE (Fig. 3*A*). Addition of EDC resulted in a shift of the protein band to higher molecular weights, representing a fraction of dimeric (lower band) and trimeric (upper band) full-length gephyrin (Schrader et al., 2004; Herweg and Schwarz, 2012; Sander et al., 2013). The band corresponding to trimer was extracted and further analyzed by mass spectrometry to identify gephyrin-βL-wt fragments. We found many overlapping peptides, covering βL residues 378–394, crosslinked to different gephyrin peptides located in the C- and E-domains (Fig. 3*B*,*C*). One particular peptide of the gephyrin E-domain (residues 329–348) served to validate our approach, since it contains residue Phe330. This residue is known to create a hydrophobic pocket together with Phe398 and Ile400 of the GlyR βL that is required for their interaction (Kneussel et al., 1999; Kim et al., 2006). Another peptide of the gephyrin E-domain was crosslinked to an N-terminal fragment of the βL (residues 378–390). Given the increase in gephyrin occupancy on attaching these residues to βL-Core (residues 394–413; Fig. 2*A*,*D*), our finding supports an extended interaction interface of the βL N-terminal sequence (βL-LO) and gephyrin. Interestingly, in addition to the E-domain, several peptides of the C-domain were crosslinked to N-terminal βL residues (Fig. 3*B*,*C*).

Based on our results, we established a model of full-length trimeric gephyrin interacting with the the GlyR β-loop (Fig. 3*C*,*D*) using a previously reported structural model of gephyrin (Belaidi and Schwarz, 2013). In the absence of structural information of the βL-extensions, we indicated the proposed position of residues 378–397 (green) and 411–426 (blue) with dashed lines, according to the identified peptides (Fig. 3*D*). Crosslinked peptides in the gephyrin E-domain (Fig. 3*C*,*D*, highlighted in purple) undergo close contacts with the N-terminal flanking region of the β-loop (shown in green), in addition to the core sequence (shown in red). Due to the localization of the peptides along the surface of the E-domain, we propose an elongated binding pocket on gephyrin occupied by N-terminal residues of the GlyR β-loop (Fig. 3*D*).

High-affinity gephyrin binding was only restored following the attachment of C-terminal β-loop residues (414–426). However, we could not identify a contact site of these residues on gephyrin with our crosslinking approach. The C-terminal region is therefore likely to shape the binding of the β-loop to gephyrin without participating in the interaction itself.

### The GlyR β-loop C-terminal 3_10_-helix is required for high-affinity binding to gephyrin

**Figure 4.**
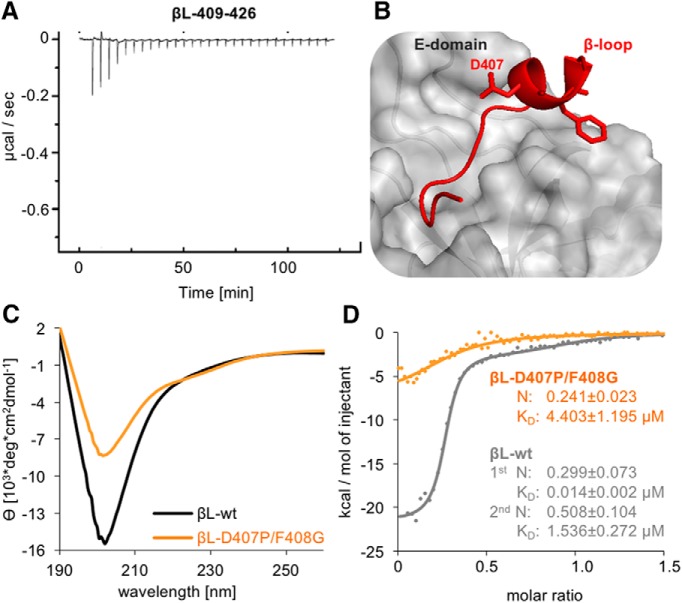
Impact of the GlyR β-loop conformation on gephyrin binding. ***A***, ITC titration profile of βL^409–426^ (445 µM) into gephyrin (25 µM). ***B***, The GlyR β-loop in association with GephE adopts a short 3_10_-helix formed by residues 406–410 (Kim et al., 2006). Two residues, Asp407 and Phe408, were mutated to proline and glycine (βL-D407P/F408G) to block the formation of the 3_10_-helix. ***C***, βL-wt and βL-D407P/F408G (both 0.21 mg/ml) folding was compared by CD spectroscopy. The mean residue ellipticity (θ) was plotted against the respective wavelength. ***D***, Comparison of the binding isotherms of representative measurements using 327 µM βL-wt peptide (gray dots, same data as in Fig. 1*D,E*) and 315 µM βL-D407P/F408G (orange dots) with 29 or 32 µM gephyrin, respectively. Curve calculation was performed based on a two-site model for βL-wt (gray line) and a one-site model for βL-D407P/F408G (orange line). Data were compared using an unpaired two-tailed *t* test: *p* = 0.0267 *K*_D_ of βL-wt high-affinity site *n* = 3 versus βL-D407P/F408G *n* = 4.

To exclude a direct interaction of the C-terminal flanking sequence with gephyrin, we tested a fragment spanning residues 409–426 in an additional ITC experiment with gephyrin (Fig. 4*A*). Only residual heat release could be detected, indicating that it is the fusion of these residues to βL-Core that turns the fragment into a high-affinity binding peptide (Fig. 2). An increase in gephyrin-binding affinity of the core sequence could therefore be a consequence of conformational changes initiated by the C-terminal flanking sequence of the β-loop. The crystal structure of the GephE dimer in complex with the β-loop showed a helical conformation between residues 406 and 411 (Fig. 4*B*) with features of a 3_10_-helix (Kim et al., 2006).

Purified βL-wt was subjected to structural analysis via CD spectroscopy, and the resulting mean residue ellipticity was plotted against the respective wavelength (Fig. 4*C*). The typical shape of a 3_10_-helix CD spectrum with minima at 208 and 220 nm confirmed the presence of this secondary structure element of βL-wt in solution (Biron et al., 2002). The βL-wt peptide contains residues that were found to be highly abundant in 3_10_-helices (Karpen et al., 1992). to interfere with the helical conformation, Asp407 and Phe408 were exchanged to Pro and Gly, respectively, both being less frequent in 3_10_-helices. CD spectroscopy of the βL-D407P/F408G peptide showed a decrease in the intensity of the 208-nm minimum, confirming that the secondary structure in βL-D407P/F408G was affected (Fig. 4*C*). The direct comparison of the ITC binding curve with βL-wt showed a reduction in heat release on titration to gephyrin (Fig. 4*D*; Table 3). Moreover, the resulting raw data could only be fitted to a one-site binding model (Fig. 4*D*). The obtained thermodynamic binding data for βL-D407P/F408G revealed a significant reduction in binding affinity (*p* = 0.0267)^i^, accompanied by a residual binding stoichiometry (Fig. 4*D*). This result suggests that the introduced mutations have a strong impact on the GlyR β-loop conformation, thus impacting the binding to gephyrin. Furthermore, the binding parameters of βL-D407P/F408G show an additional loss of low-affinity interactions with gephyrin, strengthening the notion that both binding sites depend on one another.

### Impact of high- and low-affinity binding on GlyR diffusion trapping at synapses

Our ITC experiments showed that N- and C-terminal flanking sequences of the GlyR β-loop core region are responsible for the low- and high-affinity interaction with gephyrin, respectively. To explore the effects of bimodal binding on the physiological behavior of GlyRs at synaptic gephyrin clusters, we made use of SPT of GlyR β-loop constructs using PALM in living neurons. This approach is based on the sequential stochastic conversion of photo-switchable fluorophores to follow the movement of single molecules in subsequent image frames (sptPALM; Manley et al., 2008). The tracking of fluorophores at high spatial (∼50 nm in our experiments) and temporal resolution (15 ms) gives access to diffusion parameters that reflect the strength of molecular interactions within specific membrane compartments (Specht et al., 2011).

**Figure 5.**
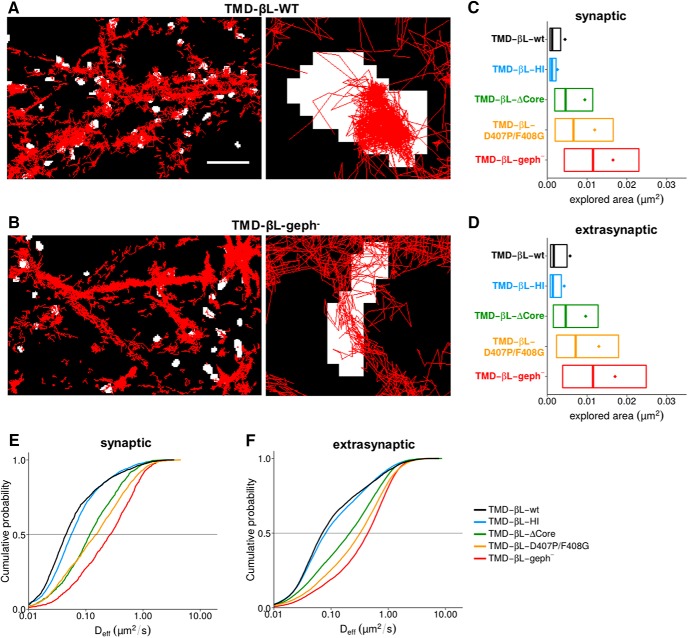
Membrane diffusion of TMD-βL variants in spinal cord neurons. ***A***, ***B***, sptPALM was done using Dendra2-tagged TMD-βL variants in cultured neurons as described in Materials and Methods. Single molecule trajectories were recorded in 10,000 frames at an acquisition rate of 15 ms (red traces). Active synapses were identified using FM 4-64 labeling (binarized fluorescence images shown in white). Left, High-density sptPALM of dendritic segments expressing TMD-βL-WT (***A***) or the gephyrin binding-deficient construct TMD-βL-geph^-^ (***B***). Right, Zoomed recordings showing confinement of TMD-βL-WT at synapses (***A***) as opposed to the high mobility of TMD-βL-geph^-^ (***B***). Scale bar: 5 µm (left panels); pixel size of FM-labeled synapses: 160 nm (right panels). ***C***, ***D***, Comparison of the areas explored by the TMD-βL variants at synapses (***C***) and in the extrasynaptic compartment (***D***), represented by the mean value (colored dots), the median, 25% and 75% quartiles of the trajectories (boxes). Explored areas were normalized by the number of detections for each trajectory. Data were compared via one-way ANOVA (Kruskal–Wallis test) followed by a *post hoc* Dunn’s multiple comparison test: all pairs were significantly different from one another with *p <* 0.001. ***E***, ***F***, Cumulative histogram of diffusion coefficients of TMD-βL variants in spinal cord neurons. Diffusion coefficients at synapses vary according to the strength of βL-gephyrin binding (***E***). The variants display comparable diffusion behaviors at extrasynaptic locations (***F***). Data were compared with one-way ANOVA (Kruskal–Wallis test) followed by a *post hoc* Dunn’s multiple comparison test: for all pairs, D_eff_ was significantly different with *p <* 0.001, except for TMD-βL-ΔCore versus TMD-βL-D407P/F408G at synapses with *p >* 0.05 (median values and quartiles with statistical comparison are given in Tables 1, 4).

We first compared the diffusion of membrane proteins consisting of β-loop sequences attached to a single TM and an extracellular Dendra2 fluorophore, thus acting as random scanners of the neuronal membrane (Specht et al., 2011). The wild-type TMD-βL-wt construct was strongly confined at synaptic gephyrin clusters, as judged by the accumulation of trajectories within FM 4-64 positive membrane domains (Fig. 5*A*). In contrast, a β-loop variant (TMD-βL-geph^-^) in which gephyrin-binding was abolished by the introduction of the point mutations F398A/I400A (Kim et al., 2006) diffused more freely in the synaptic membrane (Fig. 5*B*). To quantitatively compare the diffusion behavior of a range of high- and low-affinity β-loop variants, we calculated the membrane areas explored by TMD-βL-wt, TMD-βL-HI, TMD-βL-D407P/F408G, TMD-βL-geph^-^, as well as the construct TMD-βL-ΔCore in which the core binding domain was deleted (Fig. 5*C*,*D*; Table 4). For each trajectory, the area of the convex hull around the detections (normalized by the number of detections) was used as a measure of mobility (Renner et al., 2017). As an alternative approach, we derived the D_eff_ from the mean squared displacement (MSD) plotted against time (Fig. 5*E*,*F*; Table 4). The average number of synapses was not significantly different between the five constructs (Table 5, *p* > 0.05)^j^. However, their diffusion properties at synapses differed strongly. Explored areas and D_eff_ of TMD-βL-ΔCore at synapses were substantially larger than those of TMD-βL-wt, but smaller than those of TMD-βL-geph^-^ (Fig. 5*C*,*E*, areas *p* < 0.001, D_eff_ *p* < 0.001)^k^, confirming the presence of gephyrin-binding sequences besides the core binding pocket (Fig. 3). Similarly, the synaptic diffusion parameters of the low-affinity interactor TMD-βL-D407P/F408G were larger than those of TMD-βL-wt and TMD-βL-HI (Fig. 5*C*,*E*, areas *p* < 0.001, D_eff_ *p* < 0.001)^l^. This observation is in line with the biochemical data and demonstrates that the perturbation (TMD-βL-D407P/F408G) of the high-affinity interaction reduces the confinement of the membrane construct at synapses. Nonetheless, the TMD-βL-D407P/F408G retains a low affinity for gephyrin, as shown by the fact that its mobility was below that of the binding-deficient construct TMD-βL-geph^-^ (areas *p* < 0.001, D_eff_ *p* < 0.001)^m^. Conversely, the absence of the low-affinity site in TMD-βL-HI caused a significant acceleration compared to the wild-type (D_eff_ *p* < 0.001)^n^. These data show that low-affinity interactions participate in the slowdown of β-loop constructs at synaptic gephyrin clusters. We observed similar changes in the mobility of all the variants outside of synapses (Fig. 5 *D*,*F*; comparison of all areas *p* < 0.001 and D_eff_ *p* < 0.001)^o^, indicating that binding processes between the β-loop and gephyrin also occur in the extrasynaptic area (Ehrensperger et al., 2007). Next, we investigated possible contributions of the low- and high-affinity gephyrin binding sites of the β-loop on the synaptic and extrasynaptic mobilities of functional GlyRs.

**Table 4.** Diffusion analysis of synaptic and extrasynaptic TMD-βL variants and full-length GlyRβ subunits in rat spinal cord neurons

Parameter	β-Loop variant	Synaptic^8^ Q1/Q2/Q3	Extrasynaptic^9^ Q1/Q2/Q3
Explored area [10^−3^ µm^2^]^1^	TMD-βL-wt^3^	0.67/1.23/3.35	0.82/1.63/4.96
	TMD-βL-HI^4^	0.64/1.16/2.21	0.75/1.39/3.53
	TMD-βL-βL-ΔCore^5^	1.86/4.60/11.42	1.51/4.63/12.77
	TMD-βL-D407P/F408G^6^	1.97/6.58/16.56	2.33/7.13/17.92
	TMD-βL-geph^−^^7^	4.27/11.50/23.03	3.86/11.49/24.85
D_eff_ [µm^2^ * s^−1^]^2^	TMD-βL-wt^3^	0.02/0.05/0.11	0.04/0.07/0.26
	TMD-βL-HI^4^	0.03/0.06/0.12	0.04/0.08/0.30
	TMD-βL-ΔCore^5^	0.05/0.12/0.30	0.06/0.21/0.57
	TMD-βL-D407P/F408G^6^	0.05/0.15/0.40	0.10/0.32/0.73
	TMD-βL-geph^−^^7^	0.09/0.27/0.62	0.15/0.44/0.86
Parameter	GlyRβ variant	Synaptic (thresholded)^8^ Q1/Q2/Q3	Extrasynaptic (thresholded)^9^ Q1/Q2/Q3
Explored area [10^−3^ µm^2^]^1^	GlyRβ-wt^10^	0.54/0.73/1.05	0.60/0.91/1.76
	GlyRβ-HI^11^	0.55/0.77/1.18	0.63/0.96/1.88
	GlyRβ-LO^12^	0.58/0.83/1.40	0.77/1.47/3.04
	GlyRβ-D407P/F408G^13^	0.60/0.86/1.52	0.78/1.52/3.13
	GlyRβ-geph^−^^14^	0.84/1.51/2.73	0.92/1.83/3.48
D_eff_ [µm^2^ * s^−1^]^2^	GlyRβ-wt^10^	0.03/0.04/0.06	0.03/0.05/0.11
	GlyRβ-HI^11^	0.03/0.04/0.07	0.03/0.05/0.11
	GlyRβ-LO^12^	0.03/0.04/0.09	0.04/0.09/0.19
	GlyRβ-D407P/F408G^13^	0.03/0.04/0.09	0.04/0.09/0.20
	GlyRβ-geph^−^^14^	0.04/0.09/0.19	0.05/0.11/0.22

Median values (Q2), 25% (Q1), and 75% (Q3) quartiles of the explored trajectory areas and D_eff_ from >700 synaptic trajectories and >7000 extrasynaptic trajectories for each construct.

^1^Explored area (in 10^−3^ µm^2^).

^2^D_eff_ (in µm^2^/s).

^3^GlyR β-loop (residues 334–455) C-terminally fused to TMD and Dendra2.

^4^GlyR β-loop with deletion Δ378–393.

^5^GlyR β-loop with deletion Δ397–410.

^6^GlyR β-loop with substitution D407P and F408G.

^7^GlyR β-loop with substitution F398A and I400A.

^8^Values for synaptic trajectories.

^9^Values for extrasynaptic trajectories.

^10^mEos4b-tagged full-length human GlyRβ subunit.

^11^Full-length GlyRβ with deletion Δ378–393.

^12^Full-length GlyRβ with deletion Δ414–426].

^13^Full-length GlyRβ with substitution D407P and F408G.

^14^Full-length GlyRβ with substitution F398A and I400A. For the full-length GlyRβ variants, a threshold of 0.02 µm^2^/s was applied (corresponding to the median D_eff_ of GlyRβ-wt in fixed samples). The given Q1, Q2 and Q3 values only represent the trajectories above this threshold.

**Table 5.** Quantification of synapses per analyzed region in sptPALM recordings of TMD-βL variants in rat spinal cord neurons

β-Loop variant	Experiment 1^1^	Experiment 2^1^	Experiment 3^1^	Average^2^
TMD-βL-wt^3^	53 ± 15	48 ± 28	53 ± 0	52 ± 15
TMD-βL-HI^4^	66 ± 39	45 ± 16	100 ± 23	64 ± 31
TMD-βL-ΔCore^5^	64 ± 20	32 ± 1	85 ± 91	61 ± 45
TMD-βL-D407P/F408G^6^	37 ± 4	68 ± 6	48 ± 12	50 ± 15
TMD-βL-geph^−^^7^	55 ± 13	107 ± 35	35 ± 8	72 ± 40

Average number of synapses per experiment and for all experiments.

^1^Mean number of synapses per analyzed region and SD for the stated experiment (1, 2, or 3).

^2^mean ± SD of synapses for all three experiments.

^3^GlyR β-loop (residues 334–455) C-terminally fused to TMD and Dendra2.

^4^GlyR β-loop with deletion Δ378–393.

^5^GlyR β-loop with deletion Δ397–410.

^6^GlyR β-loop with substitution D407P and F408G.

^7^GlyR β-loop with substitution F398A and I400A.

### Diffusion behavior of high- and low-affinity variants of full-length GlyR complexes

Full-length GlyRs are pentameric complexes composed of α and β subunits. Recent quantitative PALM analyses of heteromeric GlyRs point to an α_3_:β_2_ stoichiometry in spinal cord neurons (Patrizio et al., 2017). This means that two β-loops per GlyR complex can potentially interact with gephyrin, each interaction displaying either high or low affinity. to test the impact of the bimodal binding on the behavior of full-length GlyRs, we conducted sptPALM experiments with mEos4b-tagged GlyRβ subunits in dissociated spinal cord cultures.

**Figure 6.**
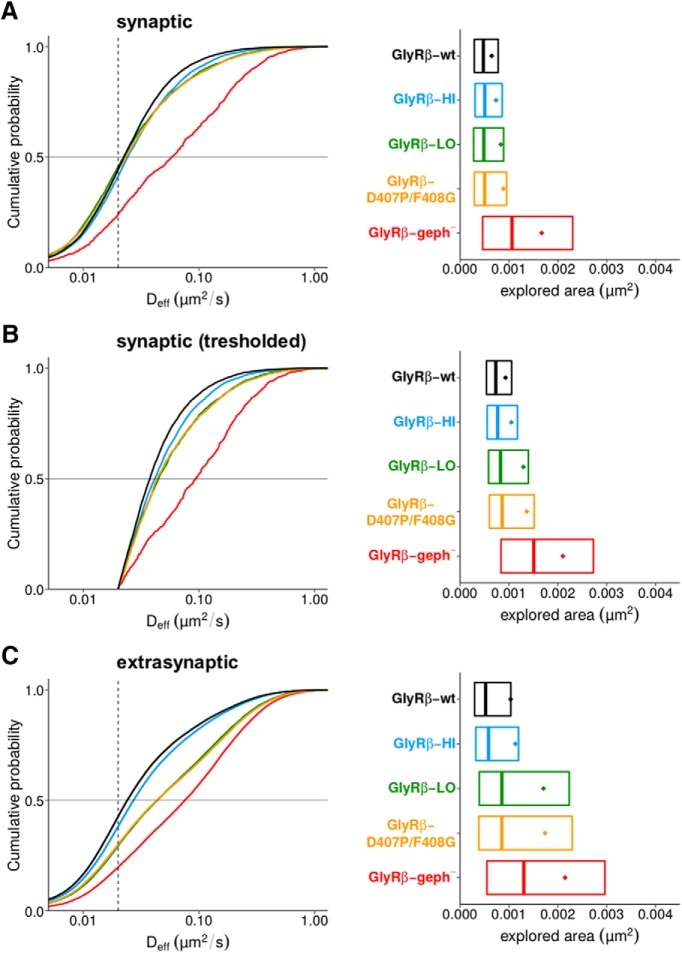
Single molecule diffusion of full-length GlyR complexes in spinal cord neurons. ***A***, D_eff_ (left panel) of mEos4b-tagged GlyRs at synapses were determined by sptPALM, using wild-type GlyRβ subunits (black trace) and the variants GlyRβ-HI (blue), GlyRβ-LO (green), GlyRβ-D407P/F408G (yellow), and GlyRβ-geph^-^ (red). The dotted line indicates the median D_eff_ value of mEos4b-GlyRβ-wt trajectories in a fixed sample (0.02 µm^2^/s) that is the limit of resolution in our recordings. The explored areas (normalized by the number of detections, right panel) are represented by their mean (colored dots), median, 25% and 75% quartiles (boxes) of the trajectory population. ***B***, Distribution of synaptic trajectories (data shown in ***A***) with D_eff_ values >0.02 µm^2^/s. Differences in the diffusion coefficients (left) and the corresponding areas (right) of the GlyRβ variants are evident for the thresholded data. Data were compared with one-way ANOVA (Kruskal–Wallis test) followed by a *post hoc* Dunn’s multiple comparison test: explored areas and D_eff_ values were significantly different for all pairs (*p <* 0.0001), except for GlyRβ-LO versus GlyRβ-D407P/F408G with *p >* 0.05 (median values and quartiles are given in Table 4). ***C***, D_eff_ (left panel) and explored areas (right) of GlyRβ variants outside of synapses (significantly different between all conditions, *p* < 0.001).

The median D_eff_ and explored areas at synapses were similar for all constructs, with the exception of the binding-deficient variant GlyRβ-geph^-^ that displayed a much higher mobility (Fig. 6*A*). However, the synaptic diffusion coefficients of the binding-competent constructs were only marginally higher than the apparent diffusion of mEos4b-GlyRβ-wt in fixed samples (Fig. 6*A*, dotted line represents the median D_eff_). On the one hand this is the result of the generally very low mobility of full-length receptor complexes at synapses due to molecular crowding (Renner et al., 2012). On the other hand, these results also show that both the low- and high-affinity sites individually contribute to a significant confinement of GlyRs at synaptic sites. To better resolve the differences in mobility, we have set a threshold for synaptic trajectories using the median D_eff_ of GlyRβ-wt in fixed samples (0.02 µm^2^/s), which constitutes the lower detection limit of our diffusion measurements (Fig. 6*B*). Under these conditions, the D_eff_ as well as the explored areas of the synaptic trajectories above the threshold were significantly larger for GlyRβ-HI, GlyRβ-LO and GlyRβ-D407P/F408G variants than those of the wild-type (D_eff_ *p* < 0.0001, areas *p* < 0.0001)^p^, indicating that both binding modes play a role in the immobilization of GlyR complexes at synaptic gephyrin clusters. What is more, the relatively small difference between GlyRβ-HI on the one hand, and GlyRβ-LO or GlyRβ-D407P/F408G on the other hand (Table 4), implies that the contribution of the low-affinity interaction may be more important than the binding studies would suggest. However, the presence of endogenous, wild-type GlyRβ subunits and the formation of mixed wild-type and mutant heteromeric complexes in infected neurons may produce intermediate effects that complicate the interpretation of these results. Outside of synapses, the pattern of GlyRβ mobility of the various constructs (Fig. 6*C*) closely matched that of the simple TMD-βL constructs (Fig. 5 *D*,*F*), confirming that the interaction between GlyRs and gephyrin also takes place at extrasynaptic membrane compartments. In addition, the data also suggest that extrasynaptic gephyrin-GlyR interactions largely depend on the high-affinity site, while synaptic interactions have significant contributions of both, the low- and high-affinity site. Taken together, our sptPALM data recapitulate the ITC binding of GlyR β-loop variants, demonstrating that the interaction of GlyRβ and gephyrin is shaped by alternative modes of binding both *in vitro* as well as in living neurons.

## Discussion

The efficiency of glycinergic signal transmission depends on the ability of gephyrin to immobilize GlyRs at synapses. The high-affinity interaction between gephyrin and the GlyR β-loop has been reported more than a decade ago (Schrader et al., 2004; Sola et al., 2004). However, the mode of interaction was found to be complex, comprising high- and low-affinity binding sites with unknown functional properties on the cellular level. Using a combination of molecular interaction studies and high-density single-particle tracking, we have elucidated the underlying molecular determinants responsible for high- and low-affinity interaction between GlyR and gephyrin and validated their contribution to the diffusion-trapping of the receptor at synapses in living cells.

The bimodal binding between gephyrin and GlyR relies on a sequence of 49 residues (378-426) in the GlyR β-loop (Schrader et al., 2004; Herweg and Schwarz, 2012). Within these 49 residues, a core binding sequence spanning residues 394–411 was shown to be critical for this interaction (Meyer et al., 1995), as confirmed by structural analysis (residues 398–410; Kim et al., 2006). Here we discovered that the core region in itself does not promote bimodal binding, but requires N- and C-terminal flanking sequences to promote low- and high-affinity binding to full-length gephyrin, respectively. During interaction, an extended binding pocket on gephyrin is occupied by the N-terminal low-affinity binding sequence of the β-loop. We concluded that the formation of the GlyR-gephyrin complex depends on additional binding sites within the E-domain as well as the C-domain of gephyrin that extend beyond the binding cleft identified in previous studies (Kim et al., 2006). Partial proteolysis of full-length gephyrin by trypsin (Herweg and Schwarz, 2012) in the presence of the β-loop provides further support to the concept that the β-loop makes contact with the C-domain of gephyrin.

The functional relevance of our findings is supported by a recent study focusing on the involvement of gephyrin in a case of Dravet syndrome (Dejanovic et al., 2015). Genetic screening identified a mutation in the gephyrin E-domain that impairs inhibitory neurotransmission (Dejanovic et al., 2015). The affected residue (G375D) is located within the extended binding pocket of the E-domain that is adjacent to the low-affinity interaction sequence of the GlyR β-loop. The reduced affinity of the β-loop for G375D gephyrin shows that mutations in the newly discovered low-affinity binding site can have direct consequences for inhibitory synaptic function.

Our data also provide evidence that high-affinity binding to gephyrin depends on the conformation of the C-terminal sequence flanking the core region of the GlyR β-loop. Despite numerous studies on the structure of the ECD or TM-domains of different LGICs (Corringer et al., 2012; Miller and Aricescu, 2014; Sauguet et al., 2014; Du et al., 2015; Huang et al., 2015), structural data of the intracellular domains (ICDs) of these receptors are largely lacking. Structural data from 5-HT3-receptor and nAchR suggest the presence of a helical structure (Unwin and Fujiyoshi, 2012; Hassaine et al., 2014) that appears to be conserved in the GlyR β-loop. We observed that the high-affinity interaction and bimodal binding of the β-loop with gephyrin is dependent on the presence of a 3_10_-helix beginning with residue 406. Truncation of the GlyR β-loop at position 411 results in a disordered C terminus (Maric et al., 2014b), supporting the notion that these residues contribute to the conformational integrity of the full-length interaction domain of the β-loop. Alternatively, replacing two critical residues of the 3_10_-helix also abolishes high-affinity binding. We therefore argue that the C-terminal flanking sequence adopts a conformation that involves the formation of a 3_10_-helix and enables the high-affinity interaction of the core-motif with gephyrin. A structural role of the ICDs has also been described in the context of the modulation of α1-subunit containing GlyRs by ethanol, G protein and interleukin-1β (Burgos et al., 2015; Patrizio et al., 2017). Given the significance of the β-loop conformation for high-affinity and bimodal binding, we thus propose to refer to this functional unit as ICD, rather than unstructured loop.

**Figure 7.**
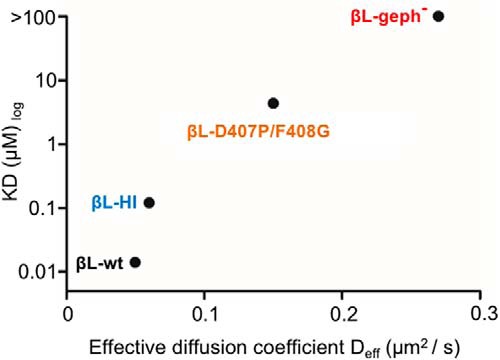
Correlation of *in vitro* binding affinities and diffusion coefficients. The binding affinities of βL-WT (high-affinity site), βL-HI and βL-D407P/F408G determined by ITC correlate with their D_eff_ obtained by sptPALM recordings in living spinal cord neurons (TMD-βL variants).

The consequences of the bimodal interaction between the β-ICD and gephyrin on the diffusion-trapping of GlyRs at synapses was assessed using single-molecule imaging in living spinal cord neurons. Making use of a TMD-βL construct that recapitulates the properties of full-length receptors in terms of cellular mobility and synaptic clustering (Specht et al., 2011; Specht et al., 2013; Masson et al., 2014), we were able to show a direct effect of high- and low-affinity interactions on the mobility of the membrane proteins. Furthermore, the relative variations of the dynamics of the GlyRβ and TMD-βL variants at synapses paralleled those in the extrasynaptic compartment, where a high proportion of GlyRs are known to be associated with gephyrin (Ehrensperger et al., 2007; Patrizio et al., 2017). We found that the binding affinities extracted from biochemical data correlate with the D_eff_ at synapses determined by sptPALM (Fig. 7). Importantly, the findings with the TMD-βL variants were reinforced by diffusion studies of full-length GlyRs containing tagged β-subunits, providing additional insight into the selective contribution of the low- and high-affinity gephyrin binding sites on synaptic and extrasynaptic receptor mobility.

According to our data, the major contribution to GlyR confinement can be assigned to the high-affinity site at both synaptic and extrasynaptic sites, with the low-affinity binding site mainly contributing to synaptic confinement. The functional relevance of the low-affinity site is evident in the sptPALM recordings of full-length GlyRs, where the mobility of GlyRβ-LO or GlyRβ-D407P/F408G at synapses was only slightly greater than that of the GlyRβ-HI variant. The low-affinity binding sites could either ensure a degree of flexibility of receptor clustering, or increase the occupancy of gephyrin clusters, or function in the finetuning of synaptic clusters. This may contribute to the dynamic equilibrium between synaptic and extrasynaptic receptors during synaptic plasticity (Specht et al., 2011) as well as the competition between GlyRs and GABA_A_Rs (Maric et al., 2011). We believe that the identified interaction domains in GlyRβ are primarily important for the binding to gephyrin and not to other synaptic proteins or cytoskeletal elements. Disruption of gephyrin binding by the exchange of two critical hydrophobic amino acids in the binding pocket (Kim et al., 2006) resulted in a further acceleration of the diffusion, exceeding the mobility of the high- and low-affinity variants. Also, the dependence of GlyR-gephyrin binding on the β-subunit was demonstrated in a reduced cellular system in the absence of endogenous components (Patrizio et al., 2017).

Full-length gephyrin forms trimers and interacts *in vitro* with isolated β-loop peptides through high- and low-affinity interactions, whereas isolated dimeric E-domains lack these properties. This is in contrast to an earlier study (Schrader et al., 2004), in which the binding of the E-domain was fitted with a two-site model. However, careful inspection of the data shows that in comparison to full-length gephyrin, the number of binding sites was ∼50% lower (Schrader et al., 2004). The results of our current study also show that only half of the binding sites in the GephE dimer are occupied, supporting the interpretation that E-domain dimerization and full receptor occupancy are mutually exclusive. Based on the trimerization and dimerization of the isolated gephyrin G- and E-domains (Schwarz et al., 2001; Sola et al., 2004; Kim et al., 2006) a hexagonal lattice beneath the postsynaptic membrane has been proposed as a clustering principle of gephyrin (Kneussel and Betz, 2000), although proof of this concept on the cellular or functional level is still missing. According to our results, a high receptor occupancy could only be reached if the E-domain dimerization within the gephyrin cluster is incomplete. Indeed, recent data have shown that the organization of gephyrin clusters is rather loose and irregular (Specht et al., 2013; Linsalata et al., 2014; Dzyubenko et al., 2016), therefore potentially providing numerous unoccupied binding sites (Patrizio et al., 2017).

Gephyrin clusters undergo a constant dynamic reorganization (Specht et al., 2013), which could allow for structural flexibility and heterogeneity of receptor binding sites within a given cluster. On average, ∼200–300 gephyrin molecules are present at synapses in cultured spinal cord neurons, with a large variability between individual synapses (Specht et al., 2013; Patrizio et al., 2017). The proportion of unbound gephyrin within the synaptic cluster determines its capacity to trap receptors (Holcman and Triller, 2006). Given the existence of high- and low-affinity interactions between gephyrin and GlyRβ, the state of gephyrin oligomerization and prior receptor occupancy could have an important impact on the recruitment of additional GlyRs at synapses. More specifically, the C-terminal flanking sequence of the β-ICD can only confer high-affinity binding to unbound gephyrin, suggesting that it plays a role in the formation of extrasynaptic GlyR-gephyrin nano-complexes that can then be added to an existing synaptic cluster. In contrast, low-affinity interactions involving the N-terminal sequences of the β-ICD can potentially occupy every free binding site. Low-affinity interactions may therefore cause a transient immobilization of unbound GlyR complexes by the synaptic gephyrin scaffold that could lead to their subsequent stabilization.

In conclusion, we have combined two different approaches to explore the binding properties of synaptic components, biochemical interaction studies using microcalorimetry and high-resolution diffusion analysis using sptPALM. Our strategy thus bridges the gap between thermodynamic binding processes analyzed *in vitro* and the diffusion-trapping of receptors in living neurons. The correlation between binding affinities and diffusion parameters implies that molecular interactions can be derived from single molecule tracking data, setting the stage for an emerging field of chemistry *in cellulo*.

## Synthesis

Reviewing Editor: Zoltan Nusser, Institute of Experimental Medicine Decisions are customarily a result of the Reviewing Editor and the peer reviewers coming together and discussing their recommendations until a consensus is reached. When revisions are invited, a fact-based synthesis statement explaining their decision and outlining what is needed to prepare a revision will be listed below. The following reviewer(s) agreed to reveal their identity: Shiva Tyagarajan, Theophilos Papadopoulos.

The reviewing editor and the reviewers agree that this is a carefully executed study that reveals important roles of the N- and C-terminal flanking sequences of the core region of the GlyR β-loop. Furthermore, it identifies new GlyR-binding sequences within the C- and E-domains of gephyrin and indicates their contribution to trapping and stabilizing of GlyRs at inhibitory synapses. The biochemical data are of high quality and the conclusions emanating from this work are far-reaching and of high interest. The results reported here advance our current understanding in the field of inhibitory synapse biology.

However, there are some issues that should be addressed before publication. In particular, additional analysis and experiments needed to do the statistics for fig 5 and 6, showing synaptic and extrasynaptic EA values. For additional, major and minor points, we below.

1. The abstract is not well structured and articulated compared to the rest of the manuscript. Reworking the abstract will enhance the reader interest in the manuscript.

2. In the significance statement (line 528) authors claim high, low affinity bound gephyrin and free state gephyrin impacting GlyR diffusion. This statement lacks clear scientific evidence based on the experimental data presented. This a bold statement that could offer fresh explanation for GlyR recruitment and organization at inhibitory synapses. However, the authors have to test this idea using full length GlyR containing mutations for high and low affinity sites, combined with sptPALM. GlyR recruitment could be co-dependent on the high and low affinity sites to create equilibrium at synapses, rather than be independent as authors have claimed in the text. In fact lack of full GlyR reconstitution experiment is a major fault line in this otherwise clean manuscript.

Alternatively, Maric et al., 2014, reported use of peptides that block GlyR interaction with gephyrin. The authors could use these peptides to block interaction and use sptPALM to study how that influences synapse recruitment and diffusion properties.

3. The sptPALM experiments add an alternative angle to this otherwise biochemical study. However, Quantifications and statistics are lacking and need to be added.

3b. The dwell time data for synaptic GlyR is missing and should be incorporated in the results.

3c. EA for synaptic and extrasynaptic GlyR should be shown separately.

4. Previous in vitro data (Sola et al., 2004) indicated that gephyrin can be 'activated' to form a protein network by lowering the pH from 8.0 to 6.7. The ITC measurements of the current study were performed at pH 8.0. Have the authors shown that lowering the pH to 7.4, i.e close to physiological conditions, does not have an impact on their ITC measurements? Did the authors check whether the E-domain alone exists as a dimer or monomer under the conditions used?

5. In page 12, lines 294-295, the authors' state: 'Addition of EDC resulted in a shift of the protein band to higher molecular weights, representing trimeric full-length gephyrin'. However, the sizes of the bands shown in figure 3A correspond rather to a dimer, not to a trimer.

6. The results shown in figures 5 and 6 are very informative but require further analysis to support the conclusions of the authors and the suggested correlation of in vitro binding affinities and diffusion coefficients. First, it is not clear whether the analysis has been performed in areas with similar numbers of synapses. By looking at panels A and B of figure 5, one has the impression that the number of active synapses visualized by FM 4-64 labelling is lower in TMD-βL-geph-expressing neurons as compared to TMD-βL-WT-expressing neurons. Here, quantification of the number of synapses per area used for the analysis would help to clarify this point. Second, the results should be accompanied by the respective statistics, as separate figure panels (i.e. diffusion coefficients, Dwell times, confinement size). This would convince the reader that the differences, for example between TMD-βL-WT and TMD-βL-HI or between TMD-βL-LO and TMD-βL-D407P/F408G shown in figure 5 are significant.

Minor points:

Line 72: The sentence is irrelevant to this basic mechanistic study.

Line 418: TMD-bL-HI caused accelerated Deff compared to wt, this is unexpected yet interesting observation. Do authors imagine a role for cytoskeleton interaction to slow GlyR diffusion? This has to be discussed clearly.

Line 524: Specht et al., reported 50-500 endogenous gephyrin molecules within a cluster. Would that help explaining how binding affinity differences can have different gephyrin molecule density within synapses?
